# Challenges of having a child with congenital anomalies in Saudi Arabia: a qualitative exploration of mothers' experience

**DOI:** 10.3389/fpubh.2023.1111171

**Published:** 2023-04-24

**Authors:** Nada Alqarawi, Sami Abdulrahman Alhamidi, Ahmed Alsadoun, Ibrahim Alasqah, Ilias Mahmud

**Affiliations:** ^1^Department of Basic Medical Sciences, Unaizah College of Medicine and Medical Sciences, Qassim University, Unaizah, Saudi Arabia; ^2^College of Nursing, King Saud University, Riyadh, Saudi Arabia; ^3^Department of Maternal and Child Health, Nursing College, King Saud University, Riyadh, Saudi Arabia; ^4^Department of Medical Surgical College of Nursing, King Saud University, Riyadh, Saudi Arabia; ^5^Department of Public Health, College of Public Health and Health Informatics, Qassim University, Al Bukayriyah, Saudi Arabia

**Keywords:** mothers of children with disabilities, parenting a child with disabilities, disability stigma, pre-school children, congenital anomalies, children with disabilities, healthcare delivery

## Abstract

**Background:**

Congenital anomalies in children lead to morbidity, mortality, or lifelong disabilities. Mothers of children with congenital anomalies face considerable obstacles in parenting their children because of their lack of knowledge about such health conditions, lack of family support, and lack of health, rehabilitation, and social care support. In Saudi Arabia, less attention are given to researching this important issue. In this context, the purpose of the study was to explore mothers' experiences and perceptions toward children with congenital anomalies.

**Methods:**

We employed a qualitative phenomenological study design. We purposively recruited 10 mothers of children with congenital anomalies from different cities in Saudi Arabia. We interviewed the mothers with an in-depth interview guideline. The interview questions were designed to explore their perception of congenital anomalies in children and their experience of parenting and seeking health, rehabilitation, education, and social care services for children with such anomalies.

**Result:**

Analysis of our data suggest that mothers of children with congenital anomalies face considerable challenges because of a lack of medical, rehabilitation, and social care support, lack of knowledge about these conditions and their management, lack of responsiveness of the healthcare providers, lack of support from the family and the stigma associated with such conditions. Consequently, mothers experience poor mental and social well-being.

**Conclusion:**

Mothers are facing considerable challenges in raising children with congenital anomalies. Regular screening for early detection of congenital anomalies, counseling support for mothers, and improving healthcare providers' responsiveness, knowledge, and skills are necessary. In addition, appropriate awareness-raising programs need to be implemented at the community level to counteract the stigma and negative attitudes of the community toward children with congenital anomalies and their families.

## Introduction

Congenital anomalies (CA) in children lead to morbidity, mortality, or lifelong disability (1, 2). The World Health Organization (WHO) (2) estimated that every year 240, 000 newborns die globally within the first 4 weeks of birth as a result of CA and associated complications. In Saudi Arabia (3), among individuals with congenital disabilities, 21% have one disability and 15% have multiple disabilities. From a longitudinal study, Kurdi et al. (4) estimated that the birth prevalence of CA is 4.12% in Saudi Arabia. The common types of CA are congenital heart disease with an estimated prevalence of 1.48%, followed by renal malformations (1.13%), chromosomal anomalies (0.27%), and neural tube defects (0.19%) (4).

The definite cause of CA is not yet conclusive. However, there are associated modifiable risk factors such as folic acid deficiency which is linked to Spina Bifida. The major factors leading to CA are genetic factors such as chromosomal abnormalities, and environmental factors such as smoking and drinking alcohol. Kurdi et al. (4) reported that major modifiable risk factors of CA in Saudi Arabia are diabetes, maternal age above 40 years, and consanguinity.

Motherhood is always challenging, and raising a child born with CA is even more challenging. Mothers of children with congenital anomalies face considerable obstacles in caring for and coping with their children's disabilities. Mothers are considered the primary providers of care to children with CA at home. However, numerous factors negatively impact the perception and experience of mothers of children with CA. For example, lack of support for mothers, poor knowledge, lack of skills, and difficulty in finding resources, and mothers' emotional responses when a child is born with CAs such as denial, feelings of guilt, worry, grief, and shame (5). These may lead to difficulty in understanding the situation, and wrong practices, which in turn may create a considerable barrier to the appropriate management of these children's conditions and delay in the appropriate medical intervention (5).

Mothers' role in promoting and enhancing the health and overall quality of life of children with CA is significant. Therefore, both children with CA and their mothers need more attention. Increased awareness, and support from the family, community, and healthcare professionals are of prime importance (6). Understanding the mothers' experience of caring for a child with CA is therefore important to improve the quality of life of both the mothers and their children with CA. In this context, the purpose of this descriptive phenomenological study was to explore the mothers' experience and perception toward their children with congenital anomalies.

## Method

### Study design

We used a qualitative approach, a descriptive phenomenological study, to explore mothers' perceptions and experiences about parenting children with CA.

### Sample and settings

We used purposive sampling to recruit participants from different parts of Saudi Arabia. The total number of participants was 10—selected from different cities (4 from Riyadh, 1 from Unaizah, 2 from Ehsaa, 1 from Rafha, 1 from Najran, and 1 from ALRean) representing different regions of Saudi Arabia. Participants were recruited *via* a social media group of mothers with children with CA. First, we sent invitations through email, including the consent form and brief details about the research aims, ethical consideration, and confidentiality, to mothers who met the inclusion criteria—mothers of children with CA aged 3–6 years.

### Demographic data of the participants

Demographic information of the 10 participating mothers and their children with CA is presented in Table 1. Mothers' age ranged between 23 and 44 years old, and mothers' age when the child was born ranged between 19 and 40 years. The lowest education was intermediate school, and the highest degree was a master. All the mothers were unemployed, except one. All participants were married. Children's age ranged between 3 and 6 years and the majority of them were male. The children's diagnoses were cleft lip and palate, developmental delays, growth delays, CHARGE syndrome, Goldenhar syndrome, Congenital heart defect, and Manitoba Trichoanal syndrome.

**Table 1:** Demographic data of the mothers.

**Age**	**Age when the child was born**	**Education level**	**Employment**	**Marital status**	**Age of the child**	**Sex of the child**	**Child's diagnosis**
23	19	Intermediate school	Unemployed	Married	4 years old	Male	Cleft lip and palate
25	19	Secondary school	Unemployed	Married	5 years old	Male	Cleft lip and palate
26	23	Secondary school	Unemployed	Married	3 years old	Male	Congenital heart defect and Cleft lip
29	23	Secondary school	Unemployed	Married	6 years old	Male	Manitoba Trichoanal syndrome and Cleft palate
31	29	Bachelor degree	Employed	Married	3 years old	Male	Cleft lip and palate
31	25	Master degree	Unemployed	Married	6 years old	Male	Goldenhar syndrome and Cleft lip and palate
32	29	Bachelor degree	Unemployed	Married	3 years old	Female	CHARGE syndrome Cleft lip and palate, developmental delays
32	29	Secondary school	Unemployed	Married	3 years old	Male	Complete cleft lip and palate
38	34	Secondary school	Unemployed	Married	4 years old	Male	Left lateral cleft lip and palate
44	40	Secondary school	Unemployed	Married	4 years old	Female	Cleft palate and growth delays

### Data collection

We did in-depth interviews in the local language, Arabic with the participants. The Zoom app was used to conduct in-depth interviews. All interviews were recorded with permission from the participants. Each interview lasted between 20 and 39 min. Data collection continued for 3 weeks. An interview guideline (Table 2) with main questions was used, and probing questions were asked as appropriate. A pilot interview was conducted before finalizing the guideline. The Interview questions have been selected based on the first interview probing related to the study's aim. All authors contributed to reviewing and finalizing the interview guidelines and plans.

**Table 2:** Interview guide.

What do you know about Congenital Anomalies?
What do you think about congenital anomalies in children? How do you view congenital anomalies in children?
Please tell us about your parenting experience with a child with CA.
Please tell us about your experience of using healthcare services for your child with congenital anomalies.
Which source do you prefer to receive health education from?
If your voice reaches the policymakers, what do you want to say to them?

### Data analysis

First, verbatim transcripts of the in-depth interviews were prepared from the audio records. We then explored and extracted the phenomenon details from participants' interviews avoiding our preconceptions. We read and reread the transcripts, then conducted open coding to extract in-depth information from the participants' perspectives. Then, we clustered the open coding to create axial coding manually. The Colazzi's (7) method—extracting significant statements (quotes) and formulating the meaning to generate themes (7) was used in the analysis. We translated the participants' quotes from the Arabic language to the English language after all authors reviewed both quotes in Arabic and English language. All authors were involved in formulating the central themes. Data started to reach saturation after the 7th interview, and no new theme emerged from the 9th interview onwards.

### Methodological rigor

To enhance the trustworthiness and rigor of the study we followed the Lincoln and Guba criteria (8). These criteria include credibility, dependability, confirmability, and transferability. All authors were engaged and reviewed all processes of this study to ensure the trustworthiness and rigor of the study findings. Moreover, prolonged engagement with the participants during the in-depth interviews and adding a couple of additional interviews after the data saturation indication following the 8th in-depth interview reinforced the credibility of the study. Furthermore, purposive sampling was used to select participants from different cities in Saudi Arabia to enhance the transferability of this study. All in-depth interviews were audio recorded. A verbatim transcript was finalized by all authors following repeated listening of the audio records to attain the dependability of this study. Bracketing was conducted to maintain the study's confirmability and avoid researchers' bias and ensure all study findings from the participants' perspectives.

### Ethical considerations

Ethical approval was obtained from the king Saud University's IRB (No.: KSU-HE-21-47). Written informed consent was obtained from all the participants before their interview. In addition, verbal consent was obtained at the beginning of each interview from the participants. Anonymity and confidentiality of information was maintained throughout the research and publication process—no identifiable personal information were required, and the interview audio records and transcript are securely kept with identifiable personal information.

## Result

### Themes

Following data analysis, we have identified four themes under the umbrella of the central theme, motherhood with extra challenges. The four themes are lack of knowledge, healthcare issues, community issues, and family issues (Table 3).

**Table 3:** Summary of the themes and subthemes.

**The central theme (motherhood with extra challenges)**	
**Themes**	**Subthemes**
1. Lack of knowledge (*n* = 10)	1-1. Terrified and shocked (*n* = 10) 1-2. Negative perception (*n* = 10) 1-3. Self-seek information (*n*=10) 1-4. Inappropriate care is provided at home (*n* = 8)
2. Healthcare issues (*n* = 10)	2-1. Lack of support and Communication issues (*n* = 10) 2-2. Not updated practice of health professionals (*n* = 7) 2-3. Lack of hospital services (*n* = 8) 2-4. Long waiting time for an appointment (*n* = 8) 2-5. Difficulty in finding supplies (*n* = 9)
3. Community issues (*n* = 10)	3-1. Bullying (*n* = 8) 3-2. Stigma (*n* = 10)
4. Family issues (*n* = 10)	4-1. Lack of support (*n* = 7) 4-2. Financial issues (*n* = 5)

#### Theme 1: Lack of knowledge (*n* = 10)

Providing care to children with CA need appropriate knowledge to implement proper care at home and manage the cases with enough information to avoid any complications, wasting time in self-help (which might be incorrect or lead to misunderstanding), and reducing mothers' stress. Within this theme, there were four subthemes—terrified and shocked, negative perception, self-seeking of information, and inappropriate care provided at home.

##### 1-1. Terrified and shocked (*n* = 10)

All the mothers indicated that they experienced shock when they first came to know their child's condition. They also mentioned some causes which led them to experience shock. The mothers complained about having no prior awareness of CA. This lack of awareness about CA is responsible for the shock they experienced when they first came to know about it from healthcare workers. In this regard, a mother stated:

“I did not have any knowledge about congenital anomalies and no awareness and nothing. It was a shock to know about it for the first time” (P 4).

Another mother experienced the same and mentioned that even the family member was shocked to discover the child's condition. She described how she felt shock when she gave birth to a child with CA and find out the case:

“I did not know it before. I think, if I knew it while the baby was inside my tummy with this problem, I would have looked for information, I would have researched, I would have understood it better, I could have consulted specialists about this ... but unfortunately, we came to know about my child's condition after I gave the birth. Following the ultrasound investigation, they [healthcare workers] said he was fine. It was a shock to know about his condition after giving birth. I became lightheaded and saw darkness around me for a moment and everyone around me was in shock too” (P 5).

##### 1-2. Negative perception (*n* = 10)

We found that mothers who experienced negative attitudes and lack of support from healthcare professionals develop a negative perception of their children with CA. A mother indicated receiving a lack of support from healthcare professionals after giving birth and during the infant's admission to a NICU, which affected her perception negatively. She stated:

“The doctors in the hospital did not reassure me at all ...did not inform me anything about the case of my child's condition and that she needs surgery to be fine ...she was admitted in a NICU for 7 months …every time they called me, they informed me of bad news about her breathing…I told myself, they are right, my daughter would die with all these congenital anomalies” (P 1).

A mother described the lack of support from the hospital staff and their negative attitude during her initial shock after delivery and the stress of child surgeries and how this impacted her psychological health negatively and produced a negative perception about further care from the same healthcare providers, a mother stated:

“This hospital destroyed my psychological health. They behaved badly with me during my labor … I will never take my son to them [for treatment] …I will take my son to people who have experience…first surgery of my son was in this hospital. I was so down when he was admitted and discharged…I was very disappointed about my son's appearance-crying, blood in his body and inflated body …any mother would be depressed ...even the fathers would” (P 5).

The mothers indicated that they associated their child's condition with a lack of support from nurses and family. In this regard, a mother stated:

“When I gave birth, they hide this thing from me, but I imagined that something bad happened … nobody reassured me or showed me a picture of him…the nursery in the hospital immediately admitted him there…only my oldest brother saw him… I asked my sister to see him and take a photo … but she said they were not allowed to enter the nursery or to take photos…after my delivery, there were suspicious things, they hide from me…I could understand from my family members' facial expressions that there is something wrong…when a nurse came to take clothes for him I asked her… she said you didn't see him… he is fine and has good health and then she left… I gave birth early in the morning and late in the evening I finally saw him. It was the most terrifying moment in my life” (P 8).

##### 1-3. Self-seeking of information (*n* = 10)

Participants indicated how they acquired information to educate themselves without guidance and support from any professionals. A mother described how she faced the situation alone and searched for information without professional help because health professionals did not provide her with essential and full information. A mother indicated:

“*Nobody told me anything although I gave birth in a good hospital … I faced all these alone, I searched by myself … nobody provided me with information*” (P 3).

A mother recommended that doctors should provide full education, and indicated that she was searching the Internet by herself for information without assistance from any healthcare professionals because nobody provided her education:

“*I feel, if a specialized doctor explained to me after birth the things would have been easier…When they told me of my child's condition, I went to search on google …I understood better when I joined the cleft lip mothers' group. Before, I did not even know the name of the condition my son had*” (P 10).

Mothers explained the issue of lack of education about their child's health condition before and after surgery. They mentioned that the healthcare professionals provided very little and no updated information to educate the family about the child's condition. The information they provided as education did not meet the level of education needed to help and support mothers. This poor health education led to her visiting the emergency department very often to help her in dealing with the case if something happened. A mother indicated:

“*I must frequently go to the emergency department; they will see the situation … There was no education for surgery from healthcare professionals. All the information I acquired through my effort. Healthcare professionals provided me with only superficial details and backdated information of my child's health condition..*.” (P 6).

##### 1-4. Inappropriate care provided at home (*n* = 8)

Most mothers indicated that they did not receive enough information and education from healthcare professionals before discharge. This led to providing inappropriate care to their children with CA at home leading to some complications and high levels of mothers' stress. In this regard a mother explained her situation when she could not provide appropriate care to her son when facing a situation at home alone:

“*I suffered from the tube because one time he (her son) removed the tube late at night, and I was in a shock …. they told me before you can re-enter the tube by yourself, but be careful, do not enter into the lung, in that case, milk will go to lung, and he will be suffocated. You know, they made me scared, I couldn't enter the tube …they said the hospital near to my home doesn't have NICU. The big hospital which has NICU and performs these procedures is 100 kilometers far from my home. I couldn't forget that night because he was hungry, starving for more than three to four hours*” (P 9).

Another mother described how lack of education upon discharge worsened the situation of her daughter due to wrong practice at home care until she learned the appropriate care from a healthcare worker:

“*How do I provide care to her, there are things upon hospital discharged, the doctor did not come… and did not tell how to provide care and medication. Nurses told me, …the way of my washing was wrong, I was afraid to use normal soap on her …I was using soap without chemicals, this thing was wrong because the inversion bladder was still open and it has to be washed with the normal soap because it sterilize more than the children's soap. The result was recurrent infection and recurrent admission within three months, I did not know until the last admission, they asked me which soap do you use? I said children's soap, and they told me this is not suitable, you must use regular soap to kills germs and bacteria. Then, they told me that this was the reason for recurrent admission*” (P 3).

Mothers realized that their care was not appropriate at home after surgery due to a lack of information. A mother stated:

“*It was wrong because …on the third day of surgery I removed the bandage, and I was sterilizing and cleaning … I was not suppose to remove the bandaged because, there was a suture which stay until the appointment …he (the doctor) was supposed to remove the bandage… I removed it before because I did not know that he (the doctor) made a third procedure (during surgery)*” (P 6).

#### Theme 2: Healthcare issues (*n* = 10)

Good quality healthcare practice and services equity to children with CA are significant and reflect on the child's health and mothers' care. Most mothers were under pressure when there was no available medical care, such as surgery, in their city. Most mothers faced difficulties getting appropriate supplies, as they lacked clear information and guidance for proper care for their children with congenital anomalies. These issues induce more challenges for mothers.

Having a child with CA is stressful particularly to make sure that the child is receiving appropriate medical and social care. In this situation, mothers need support from their family, friends, and the community. A supportive environment would ease the stress the parents particularly mothers are having. However, all the participants in this study mentioned about a lack of such support and communication issues. This second theme includes five subthemes which are, a lack of support and communication issues, not updated practice of healthcare professionals, a lack of hospital services, long waiting time for the appointment, and difficulty in finding supplies.

##### 2-1. Lack of support and communication issues (*n* = 10)

Some participants explained how they faced challenges in communication with healthcare professionals. A mother complained about doctors' negative attitude during communication and lack of support:

“Umm… there was a pediatrician, not all doctors are like that, he was negative. I was asking him a few questions about my child, he replied that's enough! you would not change anything, don't keep asking, is it big or small effects or does not affect, things just happen…. I wanted to know what the issue [my child's condition] is, but when I asked him, he answered me like that” (P 4).

While another mother explained the barriers in communication further which increase her stress and anxiety:

“When I was inside the room, a nurse approached me and gave some information, but I could not understand because she was using signs language since she could not speak Arabic … I thought my son had a problem in his throat…I was in shock and terrified … I rushed to the nursery … he was fine” (P 2).

In addition to complaining about doctors' poor communication, mothers also mentioned that the healthcare workers did not provide enough information about the treatment and prognosis of their children's condition:

“He was speaking in English and he was not interacting with us enough. He just informed us of the day of the blood test and the day of surgery. When we asked him about the surgery procedure, he told us the name of the method without explaining anything to us” (P 10).

##### 2-2. Not updated practice of healthcare professionals (*n* = 7)

Participants reported that they believe mothers of children with CA might suffer due to a lack of appropriate healthcare practices by healthcare professionals. In this regard, a mother thought that the doctor's practice and treatment plans were not appropriate to her son's case:

“*Maybe he had less experience in NICU, he (her son) was in NICU, he was supposed to have a professional feeder, they were using the wrong feeder and they were saying he didn't swallow milk…they didn't press the feeder because he was not able to suck, their method was wrong, and they put him on a tube for 12 days. When he was discharged, I discovered that their way of feeding my baby was wrong, ….”* (P 4).

The mothers indicated that doctors did not inform them about different solutions to feeding but only implemented one feeding method:

“*Doctors never told me… in all the three hospitals I visited, nobody told me to use a special feeder…. There is another better solution, why they enter the tube, it causes problems for us… I told them that they don't know this thing”* (P 1).

##### 2-3. Lack of hospital services (*n* = 8)

Inequity in hospital services negatively impacts families, particularly mothers. They suffer and face challenges such as traveling a long distance to receive the required healthcare services for their child and face the stressful situation if their child needs emergency services. Mothers complained about the lack of availability of good quality healthcare services for their child's condition in the cities they live. If any problem occurs to their child, they need to travel a long distance to receive the healthcare services:

“*In my city, the hospitals don't have special needs feeders…. they don't have the provision of special care for the children with this condition…my child and I, and his dad, we suffered a lot, they put feeding tube to my …and we give him milk by syringe, can you imagine how we put the milk in a bottle than in the syringe, then in the tube, sometimes he vomits in the tube…when the tube comes out we have to go to the hospital which is far away from our home to put another tube, we checked in our city … they don't have any services for my child”* (P 5).

Mothers complained about the lack of appropriate services, particularly NICU facilities, in their cities. They have to travel to the capital city for healthcare services:

“*No services like NICU in our city…. When I needed NICU facilities, they informed us that they don't have such facilities in their hospital, They have a delivery department without NICU facilities … they make referrals to (another hospital in the region) …. For example, when a baby is sick and needs oxygen … they don't have…. they only give basic treatment…I followed up in the capital city…Totally different …between the nearby hospital and the one I followed up (in the capital city) and … they were taking good care, good health professionals, there is no comparison at all”* (P 9).

##### 2-4. Long waiting time for appointment (*n* = 8)

Long waiting times for child's appointments for medical care increased mothers' negative experiences and increased their level of stress and anxiety. A mother complained about the long waiting time for surgery for her child. She also indicated how that long waiting time impacted her psychological health and concerns about her child's psychological health since he might face bullying:

“*… I would like my son to get the surgery done before he enters kindergarten. He will complete five years soon … but the doctor asked us to wait for their call. But, until today nobody contacted me. I tried, again and again, visiting the hospital but they told me to wait…there was no available slot for an appointment. They said they did not have time and they did not know when they would be able to call my son. They said once your turn comes, we will call you… I don't know, I feel they might have lost my contact number… it is unbelievable, I am waiting for three years”* (P 8).

Mothers believed that this long waiting time is due to issues with health insurance which affected her psychological health because she was thinking about her child's situation. A mother indicated:

“*I prepared myself to have my son cleft palate surgery, then my son become comfortable but due to health insurance there was a delay… and my psychological health was very bad…”* (P 7).

##### 2-5. Difficulty in finding supplies (*n* = 9)

Each child with CA need suitable supply to meet daily needs, mothers faced obstacles to finding the supplies, and choosing the appropriate supply for the child's case might exacerbate the child's condition and increase complication. A mother complained about the difficulty in knowing the suitable supplies for the infant case and the hospital staff did not provide this information and did not provide the essential supplies:

“*When they told my son has a cleft lip, immediately, I brought special need feeder for him but my son did not need it …I did not have the knowledge and the hospital did not provide him with special need feeder...”* (P 10).

Another mother mentioned facing difficulties when searching for an appropriate child feeder:

“*…my son has cleft lip and palate. They [healthcare workers] said that the feeder is not available here and asked us to look for it. They said we might find it in another country! The doctor drew it on a piece of paper, and I went on a search for the feeder with that drawing”* (P 4).

Mothers explained how they face difficulties when searching for appropriate child feeders, and suggested having special needs supplies available in local stores for convenience:

“*They asked me to buy a special need feeder... we were searching for it but we couldn't find it in local medical supply stores … many times we searched for it but we could not find it in our local pharmacies … if we order from the websites, it will take a long time and we don't know whether it will arrive as the same description or not… if special need feeder would have been available in our local pharmacies it would be much easier”* (P 7).

#### Theme 3: Community issues (*n* = 10)

Lack of support and lack of knowledge produces a high level of anxiety and stress among mothers. Also, the families, in particular, the mothers have a concern about their children's psychological health. The two sub-themes associated with this theme are bullying and community stigma.

##### 3-1. Bullying (*n* = 9)

Mothers described how they are anxious about their children with CA' future. They are afraid that their children might be subject to bullying in school or any social gathering. Mothers are concerned about the community's response and bullying toward their children with congenital anomalies when they would grow up with disabilities:

“*At the beginning, when I knew it was very negative thoughts and all my thinking was about the community, what would they say about my son …my son might encounter bullying”* (P 10).

Mothers expressed their concerns about community stigma regarding their children's appearance because of congenital anomalies, particularly when their children would start paying attention to their appearance:

“*I became worried thinking about what would happen when he will have to take care of himself… how would I admit him to a kindergarten. What would happen when other students would comment on his lips? I don't know, I am very worried about this issue”* (P 8).

##### 3-2. Stigma (*n* = 10)

We found that some families of children with congenital anomalies prefer to maintain the diagnosis of their children a secret to protect themselves and their children from community stigma associated with these kinds of conditions. In this regard a mother indicated that she, her mom, and her husband prefer to hide the real case of her son from other extended family members and the community to avoid facing any stigma about her child:

“*…they don't know at all that there is a problem with my son. Nobody knows, it's just me, my mother, and my husband…because the community is very harsh... our cousins would ask why this has happened to your child, and we just told them that he has only adenoids, … if I inform them further, it will open the doors for questions and questions. That's why I made it brief, people don't know these issues. Which means they don't have the education”* (P 2).

While another mother described when the community knew about their child's condition and how they are indicating any case born in the community to her child:

“*The entire community does not have a case like my child. My husband is from one tribe and I am from a different tribe. They have no background in cleft palate. The first time they knew about it was from my child's condition, now any child has a cleft lip they says, like my child, we have become the pivot for them”* (P 5).

#### Theme 4: Family issues (*n* = 10)

##### 4-1. Lack of family support (*n* = 7)

“*I suffered for a whole year alone, thinking about her, or her brother, otherwise it would be difficult....crying”* (P 3).

“*The family support gives you a positive attitude in dealing with the situation and you can bear the situation”* (P 6).

“*The first child like this, and I don't have any experience... it was very negative, in terms of my mental health, my family's psychological health, my husband's psychological health, our psychological health has deteriorated, but now it's better”* (P 5).

“*I was positive, unlike the people around me. My family was tense, afraid, worried... What are we going to do?”* (P 7).

##### 4-2. Financial issue (*n* = 5)

“*We spend lot of money in private hospitals…because my son is now 5 years old but his speech is not understandable … the governmental hospital is too far”* (P 2).

“*I am not a citizen… we are tired of health insurance. These things (congenital anomalies) are included in the insurance. However, every time we apply for approval for the insurance, they refuse... And then we filed a complaint and collected our papers to prove that they did not accept. After that time, they allowed us to do the surgery on the insurance.”* (P 7).

## Discussion

The majority of mothers in this study perceived and experienced similar types of obstacles while caring for a child with congenital anomalies. While caring for a child with congenital anomalies they experienced a lack of support (communication issues, terrified and shock, negative perception), lack of knowledge (self-seek information, inappropriate care provided at home), healthcare issues (not updated practice of health team, lack of hospital services, long waiting time for an appointment, and difficulty in finding special needs supplies), community issues (bullying and community stigma), family issues.

### Lack of knowledge

Recurrent hospital admission, long-term follow-up with healthcare professionals, long stays in NICU, and surgeries, some children with congenital anomalies need long-term medication as well as providing care daily (9–11). All of that impose tremendous stress and negative experience on mothers, particularly during the transition from hospital to home. Mothers are the primary providers of care for children with congenital anomalies at home. Mothers might experience negative experiences and considerable constraints from the time of giving birth to a child with congenital anomalies. Those mothers seek advice from different sources for information on their children's condition and prognosis. They struggle because of a lack of knowledge and instructions and skills and knowledge in case management and care. March (12) conducted a systematic literature review to explore the parents' perceptions of children with congenital heart defects during the transition period between hospital and home. Their review concluded that there are considerable concerns regarding developing appropriate medical knowledge, skills, and children feeding, and these caused significant among of stress and anxiety on their parents and impacted on children's parental relationship. Ronan et al. (6) conducted a systematic review focused on parents with children with complex health needs during the transition phase from hospital to home. They found that there is a lack of preparation and supporting parents from both hospital and community services such as inadequate discharge planning, inadequate education, and poor communication during the transition.

### Healthcare issues

To improve mothers' skills in providing care to their children with congenital anomalies at home a systematic review shed light on parents' experience of those infants having difficult issues of health when they were shifted from hospital to home. This review revealed that parents are facing obstacles during the transition from hospital to home care, stressful and emotional moments, and suffering from lack of support, particularly during the transition phase from hospital to home. They recommended that to improve the transition phase experience for mothers improved coordination, communication, and training for the parents, healthcare professionals including nurses, transition plans, policy to support the parents and their children are necessary and these will lead to positive care outcomes for both the families and their children (6).

### Lack of support

Lack of support is a major factor that might influence mothers' adaptation of care for children with congenital anomalies and their perception- either negative or positive. According to Fonseca et al. (5) who conducted a study on parenting a child with congenital anomalies and perceptions of parents about the perceived burden and personal benefits of parenting, the result revealed that mothers have higher levels of burden (negative perception ) and lower levels of personal benefits (positive perception) and predicted a higher level of parenting stress, they stated that the perception of stressor event congruent with parental adaptation with infants with congenital anomalies. They suggested comprehension evaluation on perception by healthcare professionals. They explained how to enhance parental adaptation through psychological and emotional intervention such as identifying the negative perception and modifiable factors, fostering positive perception, and reducing the negative impact on caring for children with congenital anomalies.

Moreover, issues about communication that we found in this study, have agreement with a systematic review of qualitative studies on parents' experience when they came to know that their child is born with congenital anomalies. This systematic review found that parents' dissatisfaction about how and when they are being informed about their newborn's congenital anomalies may lead to negative emotions. There is a lack of parental perspectives on how they experience the disclosure of their children's condition, particularly in a diverse population. They recommended that nurses understand people of different backgrounds such as culture and norms and interact with them accordingly. It is important to understand parents' experiences to develop appropriate communication tools, therefore, enhancing the adaptation process and coping for the mothers (13).

### Community issues

Most mothers in this study had negative emotions and anxiety. These findings agree with a quantitative study conducted to analyze and compare the level of anxiety of mothers of newborns with congenital anomalies admitted in neonatal wards in tertiary-level healthcare institutions (11). The above study found that a higher level of anxiety appears when receiving the diagnosis in the post-natal period and a moderate level of anxiety in the prenatal period. The researchers stated that anxiety levels might have an impact on mothers' emotions and this high level of mothers' anxiety might also negatively impact newborns with congenital anomalies bonding with their families (11). Furthermore, mothers in this study describe how they were concerned about the stigma in the community associated with their children's congenital anomalies and wanted to protect their children from experiencing bullying. This finding is consistent with a review article conducted in African settings regarding children with congenital anomalies and challenges (psychosocial impact, economic impact, ethical and management), they found there was a negative attitude toward the parents and the children with congenital anomalies (14). And all these are due to mystifying beliefs regarding congenital anomalies which lead to psychosocial challenges, economic challenges, and a lack of health support systems for the families and the children (14). The healthcare team also faces ethical and medicolegal challenges, and a lack of manpower and facilities to treat children with congenital anomalies (14).

### Family issues

According to different literature, mothers having children with congenital anomalies who need surgery is at higher risk of posttraumatic stress symptoms (15–17). Our findings are consistent with a qualitative study conducted to explore parents' experiences of children with congenital anomalies once knowing their children's condition. They found parents have psychological and emotional challenges toward their children with congenital anomalies. They were concerned about their care and future, traumatized by family or spouse reactions due to not understanding the children's conditions in addition to a lack of social services and support and economic obstacles (18). While another qualitative study conducted among eight families of newborns with congenital anomalies admitted to the NICU found that families were facing tremendous challenges at the beginning in providing care for their newborns with congenital anomalies (9).

However, the findings of the current study are different from past studies conducted in other countries like Ethiopia, Ghana, Iran, and Kenya (19). This implies that societies share their perceptions, experiences knowledge, and beliefs as mysterious circumstances. One of the reasons for these varied perceptions and experiences is the impact of religion, norms, traditions, and cultural lack of knowledge and experience about congenital anomalies before conceiving. One can find an overlapping opinion of mothers about congenital anomalies in the literature due to differences in life experiences and educational status.

Genetic and congenital disorders are major causes of infant mortality, morbidity, and disabilities in Saudi Arabia. Among all factors, the consanguinity (first cousin marriage) rate is found to be the most dominant factor which is 56% in Saudi Arabia (19, 20). An increase in consanguinity might have increased recessive disorder which may cause congenital disabilities (19, 20). 70% of genetically determined deafness occurs in non-syndromic form while 30% occurs in syndromic form. The most common type of genetic deafness is autosomal recessive which is ~75% in Saudi Arabia (19, 20).

Increasing parents' awareness and knowledge about congenital anomalies such as causes, treatment, and management can have a positive impact on their psychosocial health. This will also promote better practices which will ultimately positively impact parents' and children's lives. In addition, appropriate social support, and good communication between parents and healthcare professionals will facilitate effective caring and better-coping strategies. Effective cooperation between parents and health care professionals such as doctors, nurses, speech therapists, and occupational therapists will increase the parents' knowledge resulting in a low incidence of complications from children's congenital anomalies and promoting overall child health. Faddan and Ismail (21) suggested that a health education program is very crucial, particularly the role of healthcare providers in counseling and raising awareness among parents regarding congenital anomalies. Dingemann et al. (22) revealed that low maternal education correlated with a higher risk of complications among the children following surgeries for their congenital anomalies. Fontoura et al. (11) emphasized the vital role of health care professionals, particularly nurses, in protecting mothers' mental, reducing anxiety, and facilitating bonding between mothers and newborns with congenital anomalies. In addition, Pinheiro et al. (10) highlighted on the importance of health professionals' support to mothers to enhance childcare and reduce stress and anxiety. Furthermore, for the prevention of congenital anomalies among children, early detection and treatment, and management of congenital anomalies public knowledge and awareness in this regard need to be increased. Masoumeh et al. (1) highlighted the crucial impact of genetic counseling through the implementation of public health programs to raise awareness of congenital anomalies among pregnant women and the community as well. While Ronan et al. (6) recommended conducting research focusing on policy development and services development that conceptualize the support needs of healthcare professionals, parents, and children with complex needs to promote positive experiences among parents, families, and children with congenital anomalies. Therefore, the result of this study might contribute to developing policies and improving services to improve support for mothers of children with congenital anomalies in Saudi Arabia.

## Conclusion

In conclusion, the current study highlighted the stressful challenges and their influence on mothers of children with congenital anomalies. Mothers reported a lack of support, lack of knowledge of congenital anomalies, poor practices of healthcare professionals, psychological issues, stigma in the community, and lack of health and care support for children with congenital anomalies and their families. We recommend regular screening during and after pregnancy for early detection of congenital anomalies among newborns. Counseling support is necessary while informing mothers and families about their newborn's congenital anomalies. In addition to meeting the medical needs of the parents and children, healthcare providers need to focus on improving their responsiveness to the mothers and the families with a child with congenital anomalies. It is also important to formulate healthcare policies targeting children with congenital anomalies and their families. Furthermore, it is vital to understand society's attitudes, beliefs, and knowledge about congenital anomalies to promote a positive and supportive environment eliminating any stigma on this issue. In this regard, it is also crucial to bring into effective strategic plans, to raise awareness and educate people to support them to reduce the problems in the future.

## Data availability statement

The raw data supporting the conclusions of this article will be made available by the authors, without undue reservation.

## Ethics statement

The studies involving human participants were reviewed and approved by the King Saud University IRB (No.: KSU-HE-21-47). The patients/participants provided their written informed consent to participate in this study.

## Author contributions

NA, SA, and AA: conceptualization and methodology. IA, AA, and NA: formal analysis, investigation, resources, data curation, writing—original draft, and writing—reviewing and editing. SA: visualization. NA: funding acquisition. IM: writing—reviewing and editing. All authors have read and agreed to the published version of the manuscript.
